# Quality of palliative and end-of-life care: a quantitative study of temporal trends and differences according to illness trajectories in Quebec (Canada)

**DOI:** 10.1186/s12904-024-01403-9

**Published:** 2024-04-10

**Authors:** Arnaud Duhoux, Emilie Allard, Denis Hamel, Martin Sasseville, Sarah Dumaine, Morgane Gabet, Marie-Hélène Guertin

**Affiliations:** 1 Faculty of Nursing, University of Montreal, Centre-ville Station, PO Box 6128, Montréal, QC H3C 3J7 Canada; 2 Institut national de santé publique du Québec, 945 Av. Wolfe, Québec, QC G1V 5B3 Canada; 3 Centre de recherche Charles-Le Moyne (CRCLM), Campus de Longueuil - Université de Sherbrooke, 150 Place Charles LeMoyne - Bureau 200, Longueuil, QC J4K 0A8 Canada; 4 School of Public Health, University of Montreal, 7101 Av du Parc, Montréal, QC H3N 1X9 Canada

**Keywords:** PEoLC, Care access, Care equity, Quality improvement

## Abstract

**Background:**

Our aim was to assess temporal trends and compare quality indicators related to Palliative and End-of-Life Care (PEoLC) experienced by people dying of cancer (trajectory I), organ-failure (Trajectory II), and frailty/dementia (trajectory III) in Quebec (Canada) between 2002 and 2016.

**Methods:**

This descriptive population-based study focused on the last month of life of decedents who, based on the principal cause of death, would have been likely to benefit from palliative care. Five PEoLC indicators were assessed: home deaths (1), deaths in acute care beds with no PEoLC services (2), at least one Emergency Room (ER) visit in the last 14 days of life (3), ER visits on the day of death (4) and at least one Intensive Care Unit (ICU) admission in the last month of life (5). Data were obtained from Quebec’s Integrated Chronic Disease Surveillance System (QICDSS).

**Results:**

The annual percentage of home deaths increased slightly between 2002 and 2016 in Quebec, rising from 7.7 to 9.1%, while the percentage of death during a hospitalization in acute care without palliative care decreased from 39.6% in 2002 to 21.4% in 2016. Patients with organ failure were more likely to visit the ER on the day of death (20.9%) than patients dying of cancer and dementia/frailty with percentages of 12.0% and 6.4% respectively. Similar discrepancies were observed for ICU visits in the last month and ER visits in the last 14 days.

**Conclusion:**

PEoLC indicators showed more aggressiveness of care for patients with organ failure and highlight the need for more equitable access to quality PEoLC between malignant and non-malignant illness trajectories. These results underline the challenges of providing timely and optimal PEoLC.

## Background

In 2014, providing Palliative and End-of-Life Care (PEoLC) across various diseases was declared an ethical responsibility of all health care systems [1]. Adequate PEoLC is said to offer “continuity and fluidity in the continuum of services offered (…) in palliative and end-of-life care” to patients, as well as “a performance monitoring system and an evaluative approach to this palliative care continuum”. In 2015, the government of Quebec, a Canadian province, passed the Act Respecting End-of-Life Care [2], which established an expert commission mandated to examine PEoLC provided throughout the province. This commission’s first report underlined issues relating to equitable access to PEoLC, particularly for diseases other than cancer, for which prognostic is more unpredictable [2]. The commission also highlighted the need to develop and monitor PEoLC indicators to improve the assessment of the current state of PEoLC in Quebec and ultimately, access and quality of care.

In PEoLC literature, terminal decline can be categorised into four distinct trajectories related to the main cause of death: cancer, organ failure, frailty and sudden death [3]. Sudden death is often omitted, as its fast and unanticipated shift from normal function to death makes it incompatible with PEoLC delivery [4]. Thus, three illness trajectories are often addressed, each differing in the clinical profiles they serve [3, 4]. Trajectory I, associated with cancer, often follows a pattern of short and evident decline, reflecting in PEoLC. Trajectory II, associated with organ system failure, is characterized by progressive decline with an unpredictable PEoLC, punctuated by frequent and unexpected deteriorations. Finally, Trajectory III, associated with dementia and/or frailty, is characterized by a progressive disability, added to an already low cognitive and physical function.

Various indicators are used to assess quality of PEoLC on a population-level. A 2020 scoping review found that place of death and aggressiveness of care were the most frequently used indicators to assess quality of PEoLC using administrative data [5]. Monitoring place of death is valuable to identify care settings where PEoLC is most often delivered, thus orienting resource allocation [6]. Home death is frequently associated with appropriate PEoLC, yet this is contested as preferences regarding place of death vary greatly between individuals [7, 8]. Aggressiveness of care is often evaluated by assessing Emergency Room (ER) use and admissions to Intensive Care Units (ICU) in the End Of Life (EOL). Frequent ER visits and ICU admissions in the EOL are linked to poor quality of PEoLC [5, 7]. Assessing the aggressiveness of PEoLC is growing more popular as efforts are increasing to improve the efficiency of health care systems considering limited resources [5]. Moreover, many leading countries in PEoLC, such as Belgium, the United States and Sweden, monitor acute care use in the EOL to assess PEoLC quality [9].

This study aimed to fill the gap in PEoLC evaluation in Quebec by comparing quality of PEoLC provided to patients experiencing cancer (Trajectory I), organ-failure (Trajectory II) and dementia (Trajectory III) from 2002 to 2016. This is particularly relevant considering that Canadian palliative care data usually did not include Quebec data, causing a substantial under-reporting of related information [10]. To answer our objective, we analysed five PEoLC indicators; two related to place of death (percentage of home deaths and deaths in acute care beds with no PEoLC) and three related to aggressiveness of care (percentage of decedents with at least one ER visit in the last 14 days of life, percentage of decedents who visited the ER the day of death or had their death declared in the ER and percentage of decedents with at least one stay in the ICU in the last month of life).

## Methods

### Study design and data sources

This retrospective population-based study assessed healthcare services received during the last month of life in the province of Quebec from 2002 to 2016. Data on the PEoLC indicators was obtained from a study conducted and published by the Quebec’s National Institute of Public Health [11] using administrative data sourced from Quebec’s Integrated Chronic Disease Surveillance System (QICDSS). The system links 5 administrative data set [12]: (1) the health insurance registry with information on demographics and health insurance eligibility; (2) the physician claims database of all services billed to the provincial health plan; (3) the hospitalization discharge database; (4) the vital statistics death database and (5) the pharmaceutical services database, which covers prescription drug services received by Quebec residents aged 65 and older. The current study used information from the first four databases listed above and built on the Quebec’s National Institute of Public Health report [11].

### Study population

Quebec residents aged 18 or older who died between 2002 and 2016 from an illness that would have made them likely to benefit from PEoLC prior to death were included. Individuals were classified by the principal cause of death inscribed on their death certificate, consistent with the tenth International Classification of Diseases (ICD-10). Individuals were subsequently classified by illness trajectory based on the cause of death, described by Murray and al [4]. and displayed in Table 1. Specific ICD codes used to identify the population likely to benefit from PEoLC are described in detail in a previous report [11].

**Table 1:** Illness trajectories

Illness trajectories	Causes of death
**Trajectory I**	o Tumors
**Trajectory II**	o Circulatory diseases o Respiratory diseases o Endocrine, nutritional and metabolic diseases o Digestive diseases o Genitourinary diseases o Osteoarticular, muscular and skin diseases o Infectious and parasitic diseases o Congenital malformations and chromosomal abnormalities o Blood diseases, hematopoietic diseases, and certain disorders of the immune system
**Trajectory III**	o Nervous system and sensory disorders o Mental and behavioral disorders

### Measurements and definitions

Five indicators were selected; two pertaining to the place of death and three to the aggressiveness of care. *Percentage of home deaths* [1]: Home deaths were registered under a specific code in the vital statistics death database, which allowed for calculation of percentage of home deaths. *Percentage of deaths in acute care beds with no PEoLC prior to death* [2]: The vital statistics death database was first used to identify death in acute care settings, which is registered under a specific code. The hospitalization database was then used to identify hospitalisations that had ended with death and for which no sojourn in a palliative care bed was documented. *Percentage of decedents with at least one ER visit in the last 14 days of life* [3]: ER visits were identified through the physician claims database, where healthcare services are registered under a specific code when billed in the ER. ER visit in the last 14 days of life was determined when at least one healthcare service was billed in the ER. *Percentage of decedents that visited the ER the day of death or had their death declared in the ER* [4]: A visit to the ER on the day of death was determined when an ER visit date or the date of a health care service billed in the ER coincided with the date of death recorded in the vital statistics death database. When a healthcare service was billed in the ER on the day of death or an admission to the hospital was recorded in the ER, we considered that there was a contact in the ER on the day of death. *Percentage of decedents with at least one stay in the ICU visits in the last month of life* [5]: This indicator was assessed using the hospitalization database, which records the length and number of ICU stays for each hospitalization. However, the date of ICU admission is not recorded and a maximum of 3 ICU stays can be registered for each hospitalization. Thus, a visit to the ICU in the last month of life was concluded when an entire hospitalization took place in the last month of life and when at least one ICU stay was registered for this hospitalization. If the hospitalization started before the last month of life but ended in the last month of life, a visit to the ICU in the last month of life was concluded if the total number of days in the ICU was greater than the number of days hospitalized prior to the last month of life.

Information on ICD codes used to identify the study population from the underlying cause of death and the methodology, including precise codes used in the hospitalisation database or the physician billing database to compute the quality indicators are described in detail in another report [11].

### Data analysis

Descriptive analyses were performed. For each of the five indicators described above, proportions, among the population likely to benefit from PEoLC, were calculated and presented by year of death and illness trajectory. They were calculated among the population that would have been likely to benefit from end-of-life palliative care. No standardization, particularly for age, was performed.

## Results

In Quebec, between 2002 and 2016, 595,263 individuals died from an illness that would have made them likely to benefit from PEoLC prior to death. Sociodemographic characteristics and illness trajectories of these individuals are described in Table 2. These deaths represented 70.4% of all deaths declared in Quebec during this overall period (*n* = 845 596). The causes for excluding deaths were mainly trauma, sudden deaths (acute cardiovascular diseases), and infectious diseases.

**Table 2:** Sociodemographic and illness trajectories characteristics

Characteristics		N	%
*Total*		*595,263*	*100*
**Sex**	Women	305,816	51.4
	Men	289,447	48.6
**Age**	< 40	5,428	0.9
	40–49	15,741	2.6
	50–59	48,528	8.2
	60–69	94,975	16.0
	70–79	152,021	25.5
	80–89	191,827	32.2
	≥ 90	86,753	14.6
**Illness Trajectory**	I	287,290	48.3
	II	222,431	36.9
	III	88,542	14.9

Trajectory I patients accounted for nearly half (48.3%) of all decedents that would have been likely to benefit from PEoLC during the study period, whereas Trajectory II and III patients accounted for 36.9% and 14.9%, respectively. During the period from 2002 to 2016 inclusively, the yearly number of individuals that would have likely to benefit from PEoLC increased by 15.3% (*n* = 37,673 to *n* = 43,455). More precisely, the number of Trajectory I decedents saw an increase of 21.3% (17,473 to 21,188), whereas the number of Trajectory II decedents decreased by 6.3% (15,474 to 14,499). Trajectory III decedents saw the most significant growth, increasing by 64.4% (4,726 to 7,768) [11].

**Table 3:** PEoLC indicators per illness trajectories

	Population denominator	Home death [1] *		Deaths in acute care beds with no PEoLC prior to death [2] *		Visit to the ER in the last 14 days of life [3]		Visit to the ER the day of death or death declared in the ER [4]		At least one stay in the ICU visits in the last month of life [5]	
**Illness Trajectory**	N	N	%	N	%	N	%	N	%	N	%
I	287,290	25,018	8.7	79,049	27.6	117,302	40.8	34,498	12.0	17,432	6.1
II	219,431	19,823	9.1	90,379	41.3	115,333	52.6	45,772	20.9	40,208	18.3
III	88,542	5,333	6.0	112,695	14.3	18,525	20.9	5,675	6.4	3,187	3.6
Total	595,263	50,174	8.4	182,123	30.6	251,160	42.2	85,945	14.4	60,827	10.2

*excluding 977 deaths in an unknown location or outside Quebec

### Percentage of home deaths[1]

**Fig. 1:**
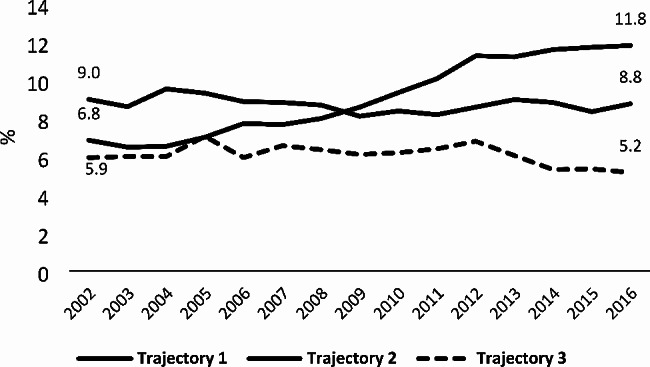
Trends in percentage of home deaths [1] regarding illness trajectories

The annual percentage of home deaths has slightly increased between 2002 and 2016, rising from 7.7 to 9.1% [10]. Overall, Trajectory I and II patients were equally more likely to die at home than their Trajectory III counterparts. Indeed, for the whole study period, 8.7% of Trajectory I decedents and 9.1% of Trajectory II decedents died at home. Trajectory III patients, of which only 6.0% died at home, were least likely to die in this setting. Between 2002 and 2016, home deaths have only increased for Trajectory II patients, rising from 6.8 to 11.8% (Fig. 1).

### Percentage of deaths in acute care beds with no PEoLC prior to death[2]

**Fig. 2:**
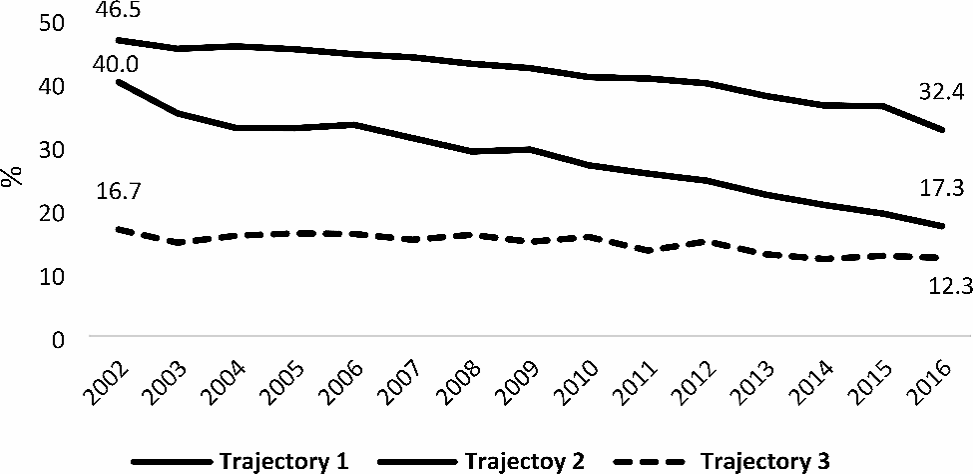
Trends in percentage of deaths in acute care beds with no PEoLC prior to death [2] regarding illness trajectories

The annual percentage of deaths in acute care settings with no PEoLC has decrease markedly from 2002 to 2016, dropping from 39.7 to 21.4% in 2016 [11]. This decrease was more important for patients dying of cancer (Fig. 2). During this overall period, death in acute care settings with no PEoLC represented 30.6% of total deaths (Table 2). The overall percentage of deaths in an acute care setting with no PEoLC was highest in Trajectory II patients (41.3%). This percentage was markedly lower for Trajectory I patients (27.6%) and lowest for Trajectory III patients (14.3%).

### Percentage of decedents with at least one ER visit in the last 14 days of life[3]

For the 2002–2016 period, 42.2% of decedents that would have been likely to benefit from PEoLC prior to death visited the ER at least once in the last 14 days of life. This percentage remained stable (42.7% in 2002 to 41.4% in 2016) [11]. Moreover, 27.8% of decedents were admitted to the hospital following an ER visit in the last 14 days of life. This percentage also remained stable (28.4% in 2002 to 28.2% in 2016) [11]. During the 2002–2016 period, ER use in the EOL was noticeably higher in Trajectory II patients [11]. Trajectory I patients were less likely to visit the ER in the last 14 days of life than their Trajectory II counterparts but were admitted to the hospital following an ER visit at a comparable rate. Trajectory III patients were least likely to visit the ER in the last 14 days of life and be admitted to the hospital following an ER visit.

### Percentage of decedents that visited the ER the day of death or had their death declared in the ER[4]

For the 2002–2016 period, 14.4% of decedents that would have been likely to benefit from PEoLC prior to death visited the ER or were admitted to the hospital from the ER, the day of their death. Over the study period, Trajectory II patients were most likely to visit the ER. In fact, approximately 1 in 5 Trajectory II decedents visited the ER the day of their death and more than 1 in 10 died or had their death declared in the ER, that may or may not include people with other types of care in the ED on that day. Trajectory I decedents were half as likely to visit the ER the day of their death and die in the ER than their Trajectory II counterparts. Trajectory III infrequently visited the ER the day of their death.

### Percentage with ICU visits in the last month of life[5]

Between 2002 and 2016, 10.2% of decedents that from PEoLC prior to death were admitted to the ICU in the last month of life. This percentage was stable over the study period, with 10.2% in 2002 to 9.5% in 2016 [11]. During this period, Trajectory II patients were markedly more likely to visit the ICU in the last month of life (18.3%) than their Trajectory I and III counterparts (6.1% and 3.6%, respectively). In fact, Trajectory I patients were 3 times less likely to be admitted to the ICU in the last month of life (6.1%) than their Trajectory II counterparts (18.3%). Trajectory III patients were rarely admitted to the ICU in the last month of life with a percentage of 3.6%.

## Discussion

This study evaluated quality of PEoLC in Quebec from 2002 to 2016, based on five key indicators. While percentage of home deaths was similar between Trajectory I and II patients, the overall percentage of home deaths remained low and only saw a minor increase between 2002 and 2016 [10]. Home deaths were infrequent for Trajectory III patients. Deaths in acute care settings, excluding palliative care settings, were decreasing. This is an encouraging result and is the result of both an increase in overall access to palliative care during hospitalization and a decrease in deaths occurring during a hospital stay [11]. Trajectory II patients scored highest in all indicators assessing aggressiveness of care, having the highest ER use and ICU admissions in the EOL. Conversely, Trajectory III patients, which are increasing in number, were least likely to visit and die in the ER or be admitted to the ICU in EOL. These results highlight the challenges of providing timely and optimal PEoLC, especially for Trajectory II decedents.

In our study, Trajectory I patients accounted for nearly half of all decedents that would have been likely to benefit from PEoLC, whereas Trajectory II and III patients accounted for 36.9% and 14.9%, respectively. This differs from other studies [13], in which different trajectories usually experience similar rates of PEoLC potential. This might be explained by the fact that some causes of deaths were excluded in our study compared to others, for instance those related to acute myocardial infarctions. Since this cause is not necessarily linked to any warning signs, the need for PEoLC is less predictable.

Surprisingly, percentage of home deaths was similar between Trajectory I and II patients. These results differ from previous studies. In fact, cancer has been shown to be a determinant of home death while cardiovascular disease has been shown to decrease the likelihood of home death [14]. In Canada, as in most countries, home death is most common in cancer patients [15]. More research is needed to understand why the percentage of home deaths is lower in Quebec. It is also important to note that we could not identify directly deaths occurring in palliative care homes (hospices). These deaths are identified as “other than home but outside hospitals” in our data. An analysis showed that this category of place of death is far more frequent for trajectory I, suggesting better access to palliative care homes (hospices) for people dying of cancer [11].

Our study also found that organ failure patients frequently use the ER in the EOL, having the most ER visits and deaths in the ER. ER is not an appropriate setting for PEoLC provision, as invasive and futile procedures are often initiated, with little consideration for patient and family care goals [16]. Care provided in the ER focuses on punctual interventions, initiated quickly to stabilize acute conditions [16]. Moreover, ER use in EOL is associated with suboptimal EOL symptom relief and elevated costs for health care systems [17]. Contrary to our results, one study set in Saudi Arabia found that ER use in the EOL tended to be higher in cancer patients (45.6%) than in organ system failure patients (29.1%) [18]. Coherent with our results, this study exposed infrequent visits to the ER in the EOL for dementia patients/frailty patients [18]. While our results show that ICU use was relatively stable over time, one study has shown that ICU in the EOL in the United-States has increased significantly in a similar time period (2000–2009), all trajectories alike [19]. In addition to being more frequently admitted to the ICU in the EOL, organ failure patients have previously been shown to receive less palliative care consultations in the ICU than patients with cancer [20]. They were also more likely to die in the ICU [20]. Quality of the EOL in organ failure patients admitted to the ICU has also been deemed the lowest [20]. Considering improving quality of life, with or without life prolongation, is unanimously preferred to the sole prolongation of life in all illness trajectories, we may question the appropriateness of the ER, and ICU, in providing PEoLC.

Organ failure patients were most likely to die in acute care settings with no PEoLC. In fact, organ failure patients are less likely to be referred to PEoLC care than patients with terminal cancer [21]. If they occur, referrals to PEoLC for organ failure patients tend to happen later in the illness trajectory, often when death is imminent [13]. Professionals may struggle to determine the right moment to refer patients with non-malignant diseases to PEoLC and lack confidence in their decision to do so [22, 23]. Insufficient professional training in PEoLC and ineffective collaboration with specialised PEoLC teams may also be at cause [23]. The combination of time of death being unforeseeable and lack of PEoLC training might explain, at least partly, not only the high number of deaths in acute care settings for Trajectory II patients, but also the high ER and ICU use in the EOL for this trajectory.

Our results corroborate previous studies, which show that patients with dementia/frailty are less likely to die at home, dying more frequently in long term care facilities [24, 25]. However, in the United-States, home deaths in dementia patients saw a significant increase between 2002 and 2014, while deaths in long term care facilities decreased [25]. Home is generally the preferred place of death [26]. Moreover, PEoLC provided in the home is argued to be more consistent and aligned with patient and family goals, ensuring quality of death [27]. Globally, results from the indicators included in this study were better for trajectory III patients. Low percentage of death in acute care settings as well as low ER and ICU use in the EOL for Trajectory III patients may be linked to dementia/frailty patients spending most of their EOL in long term care facilities. Previous studies have found an association between dementia/frailty and death in long term care facilities [28]. Nursing home expenditure is negatively associated with rates of home deaths [25]. PEoLC provided in nursing homes may be suboptimal (29), as limited resources, low staffing, high workload and lack of PEoLC training may hinder PEoLC delivery, particularly for patients with dementia. In contrast, quality of life and symptom relief have been found to be similar in nursing homes and PEoLC facilities for Trajectory III patients in one study [28]. Coherent with our results, significant increases in deaths due to dementia have been noted throughout Canada, as well as internationally [24]. In this context, more attention should be given to PEoLC provided to dementia/frailty patients in long term care facilities.

### Limits

Administrative databases and retrospective descriptive analyses cannot assess whether the actual care intensity was adequate with the patients wishes, and therefore limit the assessment of care quality to process indicators. Choice and categorization of cause of deaths in trajectories, as well as conceptualization of process indicators, may also limit the comparison with other studies. Also, analyses were not adjusted for potential confounders such as age. For a large part of patients that visited the ER on the day of their death, a death certificate was the only billed act they received during their ER visit. Therefore, these decedents could have died at home, in an ambulance or in a hospital, overestimating ER deaths and underestimating home deaths. On the other hand, any care provided in the ER that is not billed under fee for service will not be included in the calculations. Also, our results may slightly underestimate the percentage of ICU visits in the last month of life because of hospitalisations that started prior the last month of life that overlap with the last month of life. Lastly, information on care received outside hospitals was limited. For people dying of frailty/dementia, available data (SISMACQ) does not include information for services provided in long term institutions.

## Conclusion

This study suggests that lower quality of PEoLC was provided to patients with organ failure. Although results were better for patients with frailty/dementia trajectories, no data was available for healthcare delivered in long-term care facilities, where PEoLC is known to be potentially inconsistent. As hypothesized, the results of this study suggest inequities in access to quality PEoLC between malignant and non-malignant illness trajectories. This reinforces the need for better PEoLC provision for organ failure and other non-malignant illness trajectories. Future research regarding quality of PEoLC provided to dementia/frailty trajectories in long-term care facilities is also needed. Moreover, a performance monitoring system and an evaluative approach to the palliative care continuum is still needed in Quebec. More specifically, information will be important for services provided outside acute care settings, such as in long-term care institutions.

## Data Availability

Data can be available upon reasonable request to the first author of the study (AD).
